# Encapsulation of Cardamom Essential Oil in Chitosan Nano-composites: *In-vitro* Efficacy on Antibiotic-Resistant Bacterial Pathogens and Cytotoxicity Studies

**DOI:** 10.3389/fmicb.2016.01580

**Published:** 2016-10-04

**Authors:** Bushra Jamil, Rashda Abbasi, Shahid Abbasi, Muhammad Imran, Siffat U. Khan, Ayesha Ihsan, Sundus Javed, Habib Bokhari, Muhammad Imran

**Affiliations:** ^1^Department of Biosciences, COMSATS Institute of Information TechnologyIslamabad, Pakistan; ^2^Cancer Research, Institute of Biomedical and Genetic EngineeringIslamabad, Pakistan; ^3^Al-Sayed HospitalRawalpindi, Pakistan; ^4^Department of Microbiology, Quaid-i-Azam UniversityIslamabad, Pakistan; ^5^PARC Institute for Advanced Studies in Agriculture (PIASA), National Agricultural Research Centre (NARC)Islamabad, Pakistan; ^6^Industrial Biotechnology Division, National Institute of Biotechnology and Genetic EngineeringFaisalabad, Pakistan

**Keywords:** natural active agents, cardamom essential-oil, multidrug resistance, chitosan nanocapsules, MRSA, cytotoxicity

## Abstract

Natural antimicrobial agents, particularly essential oils present an excellent alternative to current antibiotics due to their potent and broad-spectrum antimicrobial potential, unique mechanisms of action and low tendency to induce resistance. However their potential as a viable therapeutic alternative is greatly compromised due to their hydrophobic and volatile nature. The objective of the current research was to explore the anti-pathogenic potential of essential oils in a bio-based nano-carrier system. Six different essential oils were tested on multidrug-resistant bacterial pathogens. However, cardamom oil was selected for nano-encapsulation because of most potent anti-microbial activity. Cardamom oil loaded chitosan nano-particles were prepared by ionic gelation method with an encapsulation efficiency of more than 90% and size was estimated to be 50–100 nm. The Zeta potential was more than +50 mV that indicate a stable nano-dispersion. Cytotoxicity analysis indicated non haemolytic and non-cytotoxic behaviour on human corneal epithelial cells and HepG2 cell lines. Cardamom oil loaded chitosan nano-particles were found to exhibit excellent anti-microbial potential against extended spectrum β lactamase producing *Escherichia coli* and methicillin resistant *Staphylococcus aureus*. Our results suggested safety and efficacy of cardamom oil loaded chitosan nano-particles for treating multidrug-resistant pathogens hence offer an effective alternative to current antibiotic therapy.

## Introduction

Community associated or hospital-acquired infectious diseases resulting from multidrug-resistant pathogens are considered leading cause of morbidity and mortality, especially in developing countries (Laxminarayan et al., 2013). The injudicious use of antibiotics is the single most important factor leading to antibiotic resistance around the world (Centres for Disease Control, and Prevention [CDC], 2013). Center for Disease Control (CDC) has endorsed the need for development of new antibiotics. However, the development of new antibiotics is expensive and time consuming. Meanwhile the genetic plasticity in pathogens results in development of resistance at a rapid rate (Centres for Disease Control, and Prevention [CDC], 2013; Lee et al., 2013). To tackle this issue there is a dire need to search for safe alternative therapies (Jamil et al., 2015).

Natural products have garnered the attention of many researchers in recent years as complementary and alternative antimicrobial agents (Jeon et al., 2011; Calo et al., 2015). Essential oils (EOs) are natural plant products comprised of complex mixtures of biologically active substances and offer potential novel template molecules and bioactive compounds (Maida et al., 2014). EOs are composed of volatile secondary metabolites having antibacterial, antifungal, anti-inflammatory, antioxidant, anticancer, and antiviral activities (Espina et al., 2011; Hossain et al., 2014; Aumeeruddy-Elalfi et al., 2015; Sharifi-Rad et al., 2015). The efficiency of the EOs depends on its chemical composition, genotypes, environmental, and agronomic conditions (Mohamed et al., 2014). Recent studies have highlighted the antimicrobial potential of EOs with few reports against multidrug-resistant bacteria (Kon and Rai, 2012; Coelho and Pereira, 2013; Nazzaro et al., 2013). Furthermore, the use of EOs as food preservatives has also been described by many to control food pathogens (Djenane et al., 2013; Perricone et al., 2015). In the pharmaceutical field, EOs have been included in the composition of many dosage forms to impart flavor and natural preservation (Mohamed et al., 2014).

Despite the excellent antimicrobial activity of EOs against pathogenic microorganisms, their utilization is very limited due to low water solubility and less stability to environmental factors including heat, moisture, and oxygen. To improve water dispersion and protect EOs from degradation, nano-sized formulations emerge as a viable solution (São Pedro et al., 2009; Donsì et al., 2011; Acevedo-Fani et al., 2015; El Asbahani et al., 2015). Moreover, nano-encapsulation improves the antibacterial activity of several antibiotics (El Asbahani et al., 2015; Jamil et al., 2016).

Chitosan (CS) has proven to be a cost effective carrier for many pharmaceutical agents (Piras et al., 2015). Major characteristics that make it an ideal carrier system are its biodegradability, biocompatibility, availability, safety, cationic charge and innate antimicrobial potential. The present work has been done to investigate the microscopic, physico-chemical and cytotoxic attributes of cardamom EOs functionalized engineered CS nanoparticles (CSNPs). Not yet reported cardamom EO potential at nano-scale to treat multidrug-resistant pathogens was also explored which may provide a bio-based alternative to overcome existing therapeutic challenges posed by multidrug-resistant pathogens.

## Materials and Methods

Cardamom, Lemon, rose, peppermint, eucalyptus, and orange EOs were purchased from local market. CS medium molecular weight was purchased from Sigma–Aldrich (Product number 448877, 75–85% deacetylation, 200–800 cP viscosity of 1% w/v in 1% v/v acetic acid). Pentasodium tripolyphospate (TPP) and glacial acetic acid were also procured from Sigma–Aldrich. All antibiotic disks were obtained from Oxoid.

### Microbial Culture Collection

All microbial cultures were collected from Al-Sayed Hospital, Rawalpindi and they were identified and characterized by standard biochemical tests and by using Analytical Profile Index (API) kit. These pathogens were preserved in glycerol broth and kept at -20°C.

Both Gram positive [Methicillin-resistant *S. aureus* (MRSA)] and Gram negative (*E. coli*) microbes were employed in the study. Resistance pattern of selected microbes was performed by Kirby Bauer disk diffusion method. Following antibiotic disks were used, Ceftazidime (CAZ 30 μg), Cefazolin (KZ 30 μg), Cefotaxime (CTX 30 μg), Imipenem (IPM), Cefepime (FEP), Cefoxitin (FOX 30 μg) Ceftriaxone (CRO 30 μg), Aztreonam (ATM 30 μg), Ampicillin (AMP 25 μg), Vancomycin (VA 30 μg), Meropenem (MEM 10 μg), and Augmentin (AMC 30 μg) and zones of inhibitions were compared with CLSI guidelines (CLSI, 2016; EUCAST, 2016).

### EO Screening for Anti-bacterial Activity

Screening of EOs to compare their antimicrobial potential was performed by agar well diffusion method. Briefly, inoculum was prepared in normal saline from pure culture and turbidity was compared to that of 0.5% McFarland solution. With the help of sterilized cotton bud microbial lawn was prepared on agar surface in all directions by rotating the plate at 90°angle. Wells were prepared on inoculated agar surface with the help of sterilized borer. Different EOs were poured in each well (50 μL). Plates were then incubated at 37°C for overnight incubation. Next day zones of inhibitions were measured. EO with the best antimicrobial activity was chosen for nano-encapsulation.

### Determination of Minimum Inhibitory Concentration (MIC)

Minimum inhibitory concentration of cardamom oil was carried out on both solid (by agar well diffusion method) and liquid media (broth dilution method). Cardamom oil was first serially diluted in different ratios with distilled water. Each dilution was poured in wells prepared on inoculated agar Petri plate. It was then incubated at 37°C. Next day highest dilution displaying zone of inhibition was marked and recorded as MIC.

Broth microdilution method was performed according to the protocol mentioned by Duarte et al. (2015) with some modifications. Briefly, 9 mL nutrient broth was sterilized in test tubes and each test tube was inoculated with 10 μL inoculum after matching turbidity with turbidity standard. Then 1 mL of each of cardamom oil dilution was added in each inoculated test tube. All test tubes were then incubated at 37°C for 18 h. After incubation highest dilution giving visibly clear broth was marked as MIC. Both positive and negative controls were also added. Negative control had only the nutrient broth and positive control had the inoculated broth (Duarte et al., 2015).

### Preparation of CSNPs

Chitosan nano-particles were prepared using ionic gelation process as described earlier (Piras et al., 2015; Jamil et al., 2016). For this purpose, two solutions were prepared. CS and tripolyphosphate (TPP) solution.

Chitosan solution was prepared by adding 0.3 g CS in 1% acetic acid solution (100 mL). It was subjected to continuous stirring until a clear solution was obtained. Whereas TPP solution was prepared by adding 0.1 g TPP in water (10 mL). TPP solution (4 mL) was added drop wise to the CS solution (100 mL) with continuous stirring. Nano-particles (NPs) formed spontaneously upon addition of TPP aqueous solution. The mixture was stirred at room temperature for 2 more hours. The NPs were then subjected to extensive ultrasonication (Cole-parmer ultrasonic processor) at 35 Hz for at least 30 min on ice bath to avoid temperature raise. It was subjected to centrifugation at 14000 *g* for 30 min at 4°C (Eppendorf 5804R Benchtop Centrifuge). For the preparation of cardamom oil loaded CSNPs, 4 mL of the cardamom oil was taken and mixed with 4 mL of TPP solution.

### Characterization of NPs

#### Scanning Electron Microscopy (SEM)

Scanning electron microscopy is a method for high-resolution imaging of surfaces. SEM analysis was done by using Jeol JSM 6490A analytical scanning electron microscope. Sample was prepared on a glass slide (1 × 1 cm) after washing it with ethanol. A tiny drop of nanodispersion was spreaded evenly over glass slide and allowed to air dry. In order to make it conductive, gold coating with Jeol Quick Auto Coater was performed (JFC-1500). The NPs were then subjected to SEM analysis under ambient conditions.

#### 3D Structure Evaluation: Atomic Force Microscopy (AFM)

While SEM) provides 2 dimensional (2D) images of sample, AFM studies were performed to get the 3 dimensional (3D) images. AFM Imaging was carried out by using Scanning Probe Microscope (Model: SPM 9600 Company: Shimadzu) in tap mode. Samples were prepared in a similar fashion as for SEM analysis without any further sample treatment.

#### Interaction and Bonding Analysis of EOs with NPs

Fourier transform infrared spectroscopy (FTIR) studies were conducted to get information about the interaction pattern and release of EOs from nano-scaffolds. For an effective drug delivery system, it is necessary that the active agent must be released slowly from the carrier moiety. If the interaction between carrier and drug is strong, drug will not be released. FTIR was conducted using Bruker-Tensor 27 Fourier transform spectrometer (Bruker Daltonik GmbH, Bremen, Germany) in order to observe the existing and newly formed functional groups between CS and EOs.

#### Determination of Zeta Potential

Nanoparticles have the natural tendency to form agglomerates due to van der Walls attractive forces dominating over repulsive forces between the particles. Zeta potential is the charge on diffused aqueous layer formed on NP surface as it is kept in water. Zeta potential was measured through Malvern zeta sizer (NanoZS) at room temperature.

#### Determination of Encapsulation Efficiency

The quantity of EO loading in CSNPs was determined by the method of Abreu with slight modifications (Abreu et al., 2012). λ_max_ was calculated for various dilutions of cardamom oil by using nanophotometer (Implen). At 295 nm (λ_max_), a clear peak was observed. Based upon readings of various dilutions, standard curve was constituted. The trend line was obtained by linear regression with standard equation (*Y* = 0.7979*X* - 1.0021) and *R*^2^ value (0.9848). Drug loaded CSNPs were isolated from the free drug by centrifugation (16,000 *g*, 25 min, Eppendorf 5415D, Eppendorf, Germany) and supernatant was quantified by spectrophotometer at λ_max_ (295 nm). The encapsulation efficiency (EE) of cardamom EO loaded CSNPs was determined using the following equation:

Encapsulation efficiency=Encapsulated EO/Total EO×100

#### Cytotoxicity Studies of NPs

These tests were done to assess the safety of NPs for *in vivo* application (Dehn et al., 2004).

### *In vitro* Hemolysis Analysis

Whole blood was collected in acid-citrate-dextrose (ACD) Vacutainer. Blood sample was mixed with the NPs suspension in 1% concentration and incubated at 37°C for 45 min. Unexposed samples (negative control), distilled H_2_O (vehicle control) and 1% SDS (positive control) were included as experimental controls. Samples were centrifuged at 14000 rpm for 5 min and absorbance of the supernatant was measured at 540 nm using Nanodrop2000 (Dobrovolskaia et al., 2008). Percentage hemolysis was calculated relative to the untreated control.

### Cytotoxicity Screening on Human Hepatocellular Carcinoma Cell Line

Human hepatocellular carcinoma cell line HepG2 (ATCC HB-8065^TM^) was sub-cultured and maintained in DMEM supplemented with fetal bovine serum (FBS) 10%, Na-pyruvate (1 mM), L-glutamine (2 mM), penicillin (100 U/mL) and streptomycin (100 μg/mL) at 37°C, and 5% CO_2_ under humidified conditions.

Potential cytotoxic effects of nanoparticles were determined via sulforhodamine B (SRB) assay as described previously (Skehan et al., 1990). In short, HepG2 cultures (10000 cells/well) were exposed to the cardamom oil loaded nanoparticles and incubated for 24 h at 37°C and 5% CO_2_. As negative control, untreated cultures were also included in each experiment. At the end of the incubation time, cultures were fixed by adding 50% pre-chilled trichloroacetic acid (TCA) and further incubated at 4°C for 1 hour. The samples were thoroghly washed with deionized water and air dried. Cells were then stained with SRB solution (0.4%) for 30 min and washed with acetic acid (1%) and dried overnight. Dried samples were photographed with a Olympus IMT-2 inverted microscope equipped with digital camera.

### Cytotoxicity Screening on Human Corneal Epithelial Cells

Human corneal epithelial (HCEC; RIKEN Bio Resource Center, Japan) cells were maintained in a mixture (ratio 1:1) of Ham’s F12 and Dulbecco’s Modified Eagle Medium (DMEM), supplemented with FBS (10%), L-glutamine (2 mM), Na-pyruvate (1 mM), penicillin–streptomycin (100 U/ml) at 37°C in a 5% CO_2_ incubator, under humidified environment. Cell harvesting was done by trypsin/EDTA (0.5 mM) at room temperature.

To analyze the toxic effects of the nanoparticles, pre-seeded HCEC cells (>90% viability; 15,000 cells/well) were exposed to the nanoparticles at 100 μg/ml for 24 h. Unexposed samples and non-cellular background (culture media only and media + NPs only) were included as experimental controls. Cell viability was assessed using MTT [3-(4,5-Dimethylthiazol-2-yl)-2,5-Diphenyltetrazolium Bromide] assay as described earlier (Mosmann, 1983). Briefly, at the end of incubation period, MTT reagent (0.5 mg/ml) was added and further incubated for 3 h at 37°C. The resulting Formazan crystals were dissolved by the addition of an equal amount of acidified 10% SDS for overnight. Absorbance was taken at 565 nm by using microplate reader (AMP PLATOS R-496) and percentage viabilities of the samples were determined relative to unexposed sample using following formula:

$$\begin{array}{l} \mathrm{Viability}(\%)=[\mathrm{Abs}(565)test sample-\mathrm{Abs}(565)\mathrm{media}+NPs only\\ /Abs(565)\mathrm{unexposed}-\mathrm{Abs}(565)\mathrm{media}]\times 100 \end{array}$$

where Abs (565) test sample and Abs (565) unexposed represent the optical density (OD) values of exposed and unexposed samples, respectively. Abs (565) media + NPs only and Abs (565) media are representatives of background OD measured for ‘media + NPs only’ and ‘media only’ samples.

### Kinetics of Bacterial Inactivation

Antibacterial activity of antibiotic loaded CSNPs was determined through standard micro-dilution broth assays. Bacterial turbidity of *E. coli* and Methicillin-resistant *S. aureus* inoculated in nutrient broth was compared to Mc-farland standards after 24 h of incubation. Ten microliters of the bacterial suspensions were added to 9 mL of sterilized broth and identical concentrations of antibiotic and nano-antibiotic formulations were added in inoculated broth and all test tubes were incubated in shaker incubator at 37°C. After 24 h OD values were measured at 595 nm on ELISA Multiplate reader. Experiment was run in triplicate and both positive and negative controls were added.

## Results

### Resistance Spectra of Selected Pathogens

In the present investigation, *E. coli* and *S. aureus* were used to determine antimicrobial efficacy of our Cardamom oil-loaded CSNPs. Both microbes were subjected to disk diffusion method to determine their resistance pattern (**Table 1**). *S. aureus* was resistant to FOX disk that is why it was called MRSA. While the *E. coli* was found to be extended spectrum β lactamase (ESBL) producer, as zone augmentation was observed between AMC disk and other cephalosporin disks. Both of the pathogens were observed to be multidrug resistant.

**Table 1:** Resistance spectra of selected microbes by disk diffusion method.

Antibiotic disk	Zone of Inhibition (mm)	
	*E. coli*	MRSA
Ceftazidime (CAZ) 30 μg	20^a^	–
Cefazolin (KZ) 30 μg	–^a^	–
Cefoxitin (FOX) 30 μg	–^a^	–
Imipenem (IPM) 10 μg	32^b^	–
Cefepime (FEP) 30 μg	17^c^	–
Ceftriaxone (CRO) 30 μg	11^a^	–
Aztreonam (ATM) 30 μg	15^c^	–
Ampicillin (AMP) 25 μg	–	–
Vancomycin (VA) 30 μg	–	20
Meropenem (MEM) 10 μg	32^b^	–
Augmentin (AMC) 30 μg	–^a^	20

*Agar Petri plates were inoculated with test organisms and antibiotic disks were applied over it. After 24 h incubation zone of inhibitions were measured in mm.*

*^a^ESBL Phenomenon was exhibited between these antibiotic discs so their exact zones cannot be interpreted.*

*Whereas MRSA, methicillin-resistant *Staphylococcus aureus*.*

*^b^Susceptible.*

*^c^-Resisatant.*

*–No zone of inhibition.*

### EO Screening and MIC Determination

Essential oils were subjected to agar well diffusion assay to determine efficacy of selected EOs against multidrug-resistant pathogens (**Table 2**). It was observed that cardamom oil was most active against both MRSA and ESBL producing *E. coli* so it was selected for encapsulation in NPs. Cardamom oil emulsion was prepared in various dilutions with distilled water. MIC was calculated to be 25 % (Percent v/v) for *E. coli* and (10 %) (Percent v/v) for MRSA.

**Table 2:** Screening of essential oils (EOs) by agar well diffusion assay.

Volatile oil	*E. coli* (mm)	MRSA (mm)
Cardamom	22	39
Rose oil	22	22
Peppermint oil	No	No
Lemon oil	No	18
Orange oil	14	12
Eucalyptus oil	14	15

*Agar plates were seeded with microbial inoculum and 50 μL EOs were poured in each well. Following 24 h incubation zones of inhibitions were measured.*

*MRSA, Methicillin-resistant *S. aureus*.*

### Characterization of NPs

**FIGURE 1:**
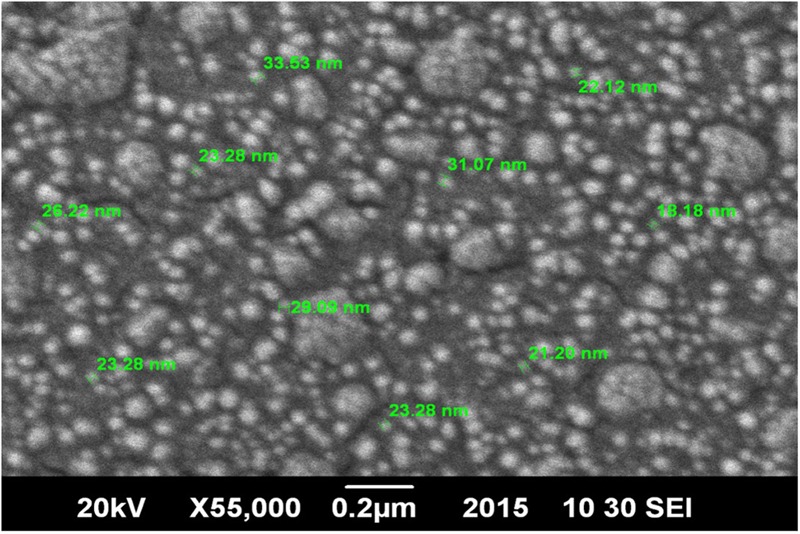
**Scanning Electron Microscope (SEM) micrographs.** Morphological evaluation of chitosan nano-particles (CSNPs) by scanning electron microscope (SEM) micrographs at 55000×.

**FIGURE 2:**
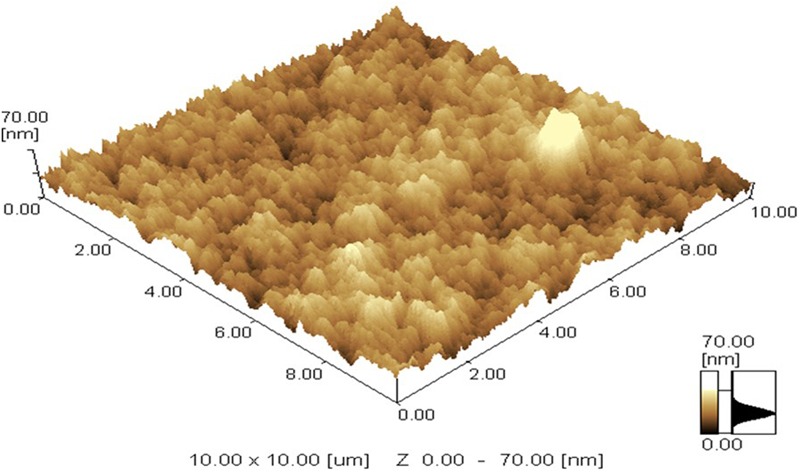
**Atomic Force Microscope (AFM) image.** AFM micrograph of CSNPs was taken at room temperature. Maximum height of 70 nm was observed.

Chitosan NPs were prepared by ionic gelation method and then subjected to various characterization techniques to ascertain their size and shape. SEM (**Figure 1**) and AFM (**Figure 2**) analysis depicted CSNPs size to be 50–100 nm with quite uniformity in both size and shape. More than 90% encapsulation efficiency of cardamom oil loaded CSNPs was observed.

### Interaction Studies of Cardamom Oil with CSNPs

**FIGURE 3:**
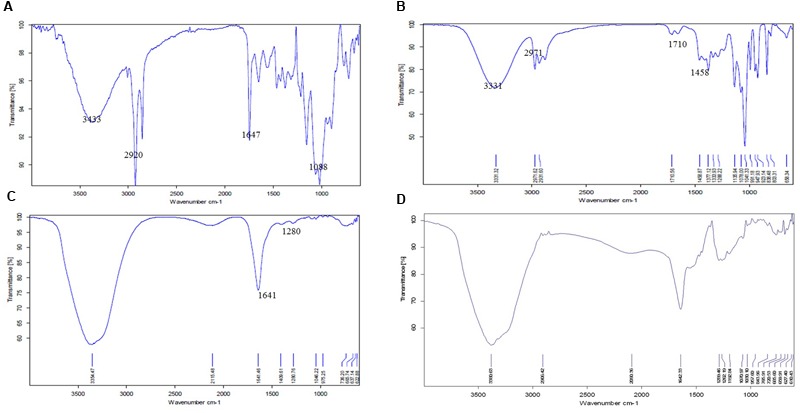
**Fourier transform infrared spectroscopic (FTIR) spectra of **(A)** CS raw material, **(B)** cardamom oil raw material, **(C)** blank CSNPs, and **(D)** cardamom oil-loaded CSNPs.** From these images it can be observed that the FTIR image of CSNPs and EOs loaded CSNPs are almost superimposable, indicating no chemical reaction between CS and EOs. The entrapment of EOs is mere physical.

Fourier transform infrared spectroscopy spectra of CS powder shows characteristic peaks at 3433 cm^-1^ (OH and NH2 stretching), 2920 cm^-1^ (CH stretching), 1647 cm^-1^ (amide I), 1088 cm^-1^ (C–O–C stretching; **Figure 3A**). Pure cardamom oil spectra displays sharp characteristic peaks at 3331 cm^-1^ (OH stretching), 2971 cm^-1^ (OH stretching), 1710 cm^-1^ (C = O stretch representing aldehyde or ketones), 1458 cm^-1^ (CH_2_ bending), 1377 cm^-1^ (CH bending), and 1333 cm^-1^ (C–N stretching; **Figure 3B**). For empty CSNPs (**Figure 3C**), the amide I (NH_2_ bending) peak shifted from 1647 to 1641 cm^-1^, and new peaks appeared at 1280 cm^-1^ (C–N stretch) implying the complex formation via electrostatic interaction between NH^3+^ groups of CS and phosphoric groups of TPP within the nanoparticles. All the above characteristic peaks also appear in the spectra of cardamom oil loaded CSNPs at almost the same wave number indicating no modification or interaction between the EO and CSNPs (**Figure 3D**).

### Stability of Cardamom Oil with CSNPs

**FIGURE 4:**
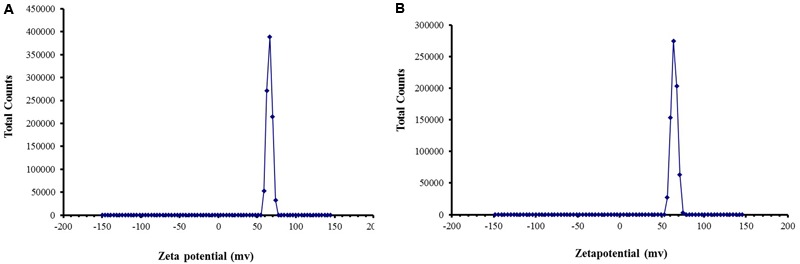
**Zeta Potential of **(A)** blank CSNPs and **(B)** cardamom oil-loaded CSNPs.** Zeta potential was measured through Malvern Instrument. A positive zeta potential of more than 50 mV was observed for both blank and EOs loaded CSNPs.

Zeta potential studies predicted long term stability as the zeta potential values were more than 50 mV in both empty and cardamom oil-loaded CSNPs (**Figure 4**).

### Cytotoxicity Studies

**FIGURE 5:**
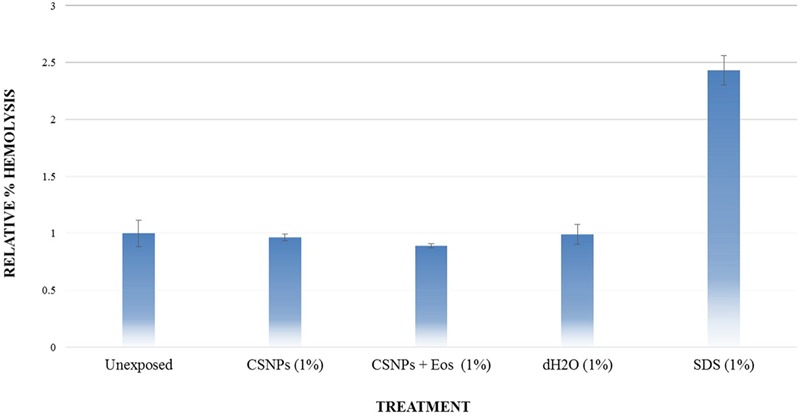
**Hemolysis analysis.** Evaluation of % hemolysis induced by chitosan NP and cardamom oil-loaded CSNPs. It can be observed that both empty and EOs-loaded CSNPs are not causing hemolysis.

**FIGURE 6:**
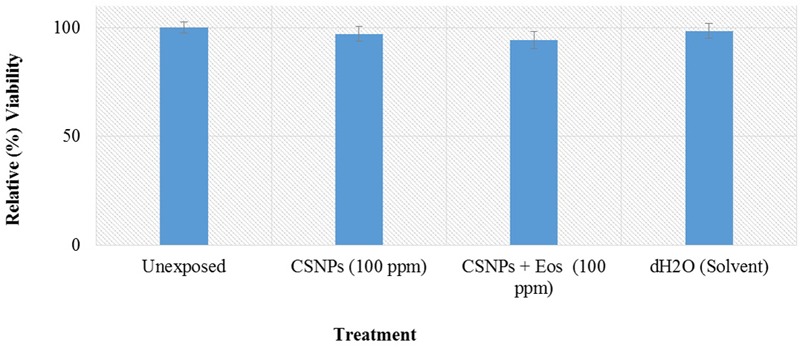
**Cytotoxicity analysis on Human corneal epithelial cells.** Growth and survival of Human corneal epithelial cells was assessed by MTT [3-(4,5-Dimethylthiazol-2-yl)-2,5-Diphenyltetrazolium Bromide] assay.

**FIGURE 7:**
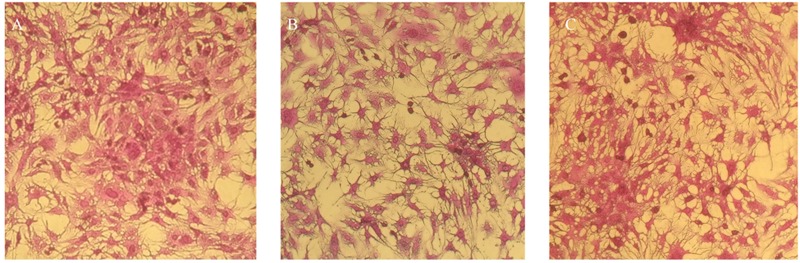
**Cytotoxicity analysis on HepG2 cancerous cell line **(A)** untreated HepG2 cells and **(B)** blank CSNPs **(C)** essential oil-loaded CSNPs.** Cytotoxicity testing was done to check the safety of NPs on human cell. It can be observed that both the blank and EOs loaded chitosan NPs were not causing any cell damage.

Potential cytotoxic effects of the cardamom oil loaded CSNPs was performed via hemolysis and potential to induce morphological alterations in mammalian cells. Both empty and EOs-loaded CSNPs did not display any hemolytic effect (**Figure 5**). Furthermore, no necrotic damage to monolayer cell lines or any change in cell morphology was observed (**Figures 6** and **7**).

### Antimicrobial Activity of Cardamom Oil-Loaded CSNPs

**FIGURE 8:**
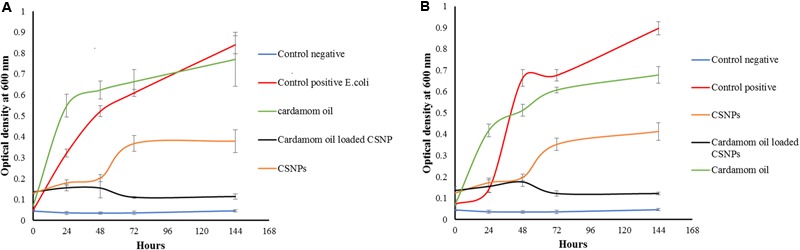
**Growth kinetics of (A) *Escherichia coli* (B) Methicillin-resistant *Staphylococcus aureus*.** It can be observed that the cardamom oil-loaded CSNPs have completely eradicated both pathogens whereas, bare CSNPs initially controlled the pathogens for 2 days after that a rapid growth was observed.

According to the results of the current study, cardamom oil alone failed to inhibit both pathogens because cardamom oil was incorporated below MIC (**Figure 8**). Both empty CSNPs and EOs-loaded CSNPs effectively controlled the growth of pathogens for first 48 h. However, after 48 h growth appeared with empty CSNPs whereas the EOs-loaded CSNPs maintained their anti-microbial potential till 7 days (**Figure 8**).

## Discussion

Essential oils of plant origin are used both domestically and industrially. According to an estimate more than 3000 EOs have been produced by different plants. However, almost 300 are important from the commercial point of view. EO market all over the world is estimated to be 700 million US$ (Raut and Karuppayil, 2014).

In the present study we encapsulated EOs in CSNPs and then their antimicrobial potential on multidrug-resistant *S. aureus* and *E. coli* was determined. The *S. aureus* was confirmed to be MRSA because it was exhibiting resistance against cefoxitin disk. The proportion of *S. aureus* isolates that are resistant to methicillin is increasing rapidly. Previously, it was considered as nosocomial pathogen. However, infections caused by oxacillin (methicillin)-resistant *S. aureus* (MRSA) are a problem not only in health care institutions but also in community as well (Klevens et al., 2006). *E. coli* used in the study was ESBL positive. The prevalence of (ESBL) positive *E. coli* is also reported to be very high. According to a study it was estimated that almost 70–90% of *E. coli* population is ESBL positive (Kumarasamy et al., 2010). ESBL positive pathogens are considered to be resistant to whole cephalosporin range and the only safe treatment for curing ESBL positive pathogens is carbapenem and now the pathogens have developed resistance against carbapenem as well. So the clinicians are left with no safe antibiotics and there is a dire need to search for new antibiotics against which microbes cannot develop resistance rapidly.

Elettaria cardamomum (cardamom, family; Scitaminaceae) is a perennial herb, indigenous to India, Pakistan, Burma, and Sri Lanka (Khan et al., 2011). In addition to its wide use for culinary purpose, cardamom has folkloric repute as carminative, stomachic, diuretic, antibacterial, antiviral, antifungal and is considered useful in treatment of constipation, colic, diarrhoea, dyspepsia, vomiting, headache, epilepsy, and cardiovascular diseases (Gilani et al., 2008). Besides cardamom oil, the antimicrobial properties of several other EOs against a number of pathogenic microorganisms involved in foodborne illness have been demonstrated in previous investigations (Roby et al., 2013; Zhang et al., 2016).

Previous studies have reported MIC of cardamom EO at 0.4% against *S. aureus* ATCC 6538 and 0.8% against *E. coli* ATCC 8739 (Gochev et al., 2012). However, in the present study we tested the EOs against multi drug resistant pathogens that is why higher MIC values were achieved as compare to previous reports.

Numerous studies have reported an enhancement of the physical and antimicrobial properties of EOs-loaded nano-emulsions as compared to their conventional emulsions (Buranasuksombat et al., 2011; Liang et al., 2012; Salvia-Trujillo et al., 2014; Severino et al., 2015). The encapsulation of eugenol and carvacrol into nanometric surfactant micelles also resulted in enhanced antimicrobial activity. However, not yet reported previously, cardamom oil was encapsulated in CSNPs to protect it and to achieve antimicrobial activity at low dose below MIC value against MDR pathogens.

Hosseini et al. (2013) reported oregano loaded CSNPs by incorporating Tween, however, in the present study, cardamom oil-loaded CSNPs were prepared directly by ionic gelation method after mixing EO with TPP. It was observed after SEM (**Figure 1**) and AFM (**Figure 2**) analysis that NPs were in the range of 50–100 nm and they were uniformly distributed. CS is a very popular material as nano-carrier and has been widely studied by numerous authors. However, varying sizes in the range of 40–500 nm have been reported previously (Abreu et al., 2012; Hosseini et al., 2013).

While studying the interaction of cardamom oil with CS scaffolds, the materials were subjected to FTIR analysis. CS powder shows characteristic peaks that were in accordance to previously reported spectra (Khan et al., 2002; Vitali et al., 2006). The results of FTIR indicated that cardamom oil might be encapsulated into the CSNPs. Evidence of EOs and CS interaction was in good agreement with previous literature (Abreu et al., 2012). The CS and EOs-loaded CSNPs were subjected to stability testing by means of zeta potential analysis. Zeta potential studies predicted long term stability as the zeta potential values were more than + 60 mV in both empty and cardamom oil loaded CSNPs (**Figure 4**). Results of this study were in good accordance to the previous data (Calvo et al., 1997; Raja et al., 2013; Chang et al., 2015). A charge of more than + 30 mV is consider as an indicator of stability as the mutual repulsive forces would be more as compare to van der walls attractive forces between the NPs. In addition to stability the positive charge on both empty and loaded CSNPs indicated good interaction with pathogens as well. Bacterial cell wall carries negative charge and our developed NPs are carrying positive charge so there is a prediction of very strong interaction.

Our results of cytotoxicity were also found to be in accordance with the results of De Campos, where it was observed that both HCEC and HepG2 cell lines after CSNPs treatment yielded 100% cells viability (De Campos et al., 2004). Hemolysis or destruction of red blood cells is a very significant aspect to be considered for *in vivo* applications of all pharmaceutical agents as it can lead to anemia, jaundice and other pathological conditions. CSNPs were found to be absolutely non-hemolytic. Our results were found to be in good accordance to the findings of Zhou et al. (2015).

The antimicrobial potential of medicinal plants is well documented. Donsì investigated the nano-encapsulation of EO and has reported enhanced antimicrobial effect against multiple pathogens causing food spoilage (Donsì et al., 2011; Aumeeruddy-Elalfi et al., 2015). Moreover, Liang also reported enhanced and prolonged antimicrobial activity of EOs after nano-encapsulation (Liang et al., 2012). However, this is the first report on nano-encapsulation of cardamom oil in CSNPs. According to our findings cardamom oil loaded CSNPs were highly effective in combating both ESBL-producing *E. coli* and MRSA. Whereas, cardamom oil alone failed to eradicate them. However, the CSNP inhibited the growth of pathogens till first 48 h after that there was a rapid increase in optical density of both pathogens.

The positive charge due to the presence of –NH^+3^ have been mentioned as important factors that influence the antibacterial activities of CS (Chang et al., 2015). The strong adhesive properties of CS with bacteria are due to these opposite charges. However, to maintain the antimicrobial potential of CS, it must be incorporated with some antimicrobial agents and naturally occurring antimicrobials are the best choice for this purpose. It serves dual purpose, protecting the EOs from degradation for prolonged antimicrobial efficacy and sustaining the innate inhibitory effect of CS to inhibit resistant superbugs.

## Conclusion

Cardamom EO-loaded CS nano-capsules were observed to be highly effective in controlling multi drug resistant *E. coli* and MRSA *in vitro* without showing any signs of toxicity to human cells.

## Author Contributions

BJ performed all research work and compiled data and wrote manuscript. RA performed cytotoxicity analysis and helped in manuscript write up. SA helped in collecting and identifying pathogens. MI helped for FTIR analysis. SK helped in studying growth kinetics studies on ELISA multi plate reader. AI helped in doing zeta potential analysis. SJ provided technical assistance in experimentation and write up. HB also provided technical assistance in experimentation and manuscript write up and MI^∗^ provided technical assistance in experimentation and compilation of data and manuscript write up.

## Conflict of Interest Statement

The authors declare that the research was conducted in the absence of any commercial or financial relationships that could be construed as a potential conflict of interest.
